# The association between total, animal-based, and plant-based protein intake and cognitive decline in older adults

**DOI:** 10.1007/s00394-025-03810-x

**Published:** 2025-10-06

**Authors:** Maud Peperkamp, Margreet R. Olthof, Marjolein Visser, Hanneke A. H. Wijnhoven

**Affiliations:** Department of Health Sciences, Faculty of Science, Amsterdam Public Health Research Institute, Vrije Universiteit Amsterdam, 1081 HV Amsterdam, The Netherlands

**Keywords:** Protein intake, Animal-based protein, Plant-based protein, Cognitive function, Cognitive decline

## Abstract

**Objective:**

Epidemiological studies have suggested a potential cognitive benefit of higher protein intake, but findings have been inconsistent and inconclusive. This study examined associations of total, animal-, and plant-based protein intake and cognitive function and decline in older adults.

**Methods:**

Data were analysed from 1,339 community-dwelling adults aged 55 + (median age 65.2 y, IQR 61.0;72.1) participating in the Longitudinal Aging Study Amsterdam. Protein intake, measured via a Food Frequency Questionnaire (2014–2015), was expressed as energy percentage (%E) from total, animal-, and plant-based sources. Cognitive domains—global cognition (MMSE), information processing speed (Coding task), episodic memory (15WT), and executive function (Word Fluency)—were measured every three years between 2011 and 2021 and converted to z-scores. Linear mixed models evaluated associations between cognitive function and decline (testing interaction by age), adjusting for confounders including diet quality. Sex interactions were tested.

**Results:**

Mean protein intake was 1.1 g/kg/d (SD: 0.3). Higher quartiles of protein intake (%E) were associated with lower episodic memory (Q2–Q4 vs Q1, β_Q4 = − 0.18 (− 0.30; − 0.06)) and faster decline in global cognition (Q3 vs Q1, β = − 0.02 (− 0.03; − 0.00)) and processing speed (Q3 vs Q1, β = − 0.02 (− 0.03; − 0.01)). Results were comparable for total protein intake in g/adjusted kg/d. Animal-based protein intake was associated with faster decline in processing speed (Q3,Q4 vs Q1, β_Q4 = − 0.01 (− 0.02; − 0.00)). Plant-based protein intake was associated with higher processing speed in females only (Q4 vs Q1, β = 0.31 (0.09;0.53)) and lower episodic memory (Q4 vs Q1, β = − 0.16 (− 0.30; − 0.02)). No associations were found for executive function.

**Conclusion:**

These findings suggest protein intake does not benefit cognitive function in older adults. Negative associations may relate to protein food sources.

**Supplementary Information:**

The online version contains supplementary material available at 10.1007/s00394-025-03810-x.

## Introduction

The increasing global ageing population has led to a proportional rise in neurocognitive disorders, including cognitive decline and dementia, now affecting approximately 50 million people worldwide [1]. Despite the high prevalence and significant negative impact on an individual and societal level, pharmacological treatment options remain limited, primarily focusing on symptom management rather than disease prevention or modification [2]. As a result, efforts have focused on prevention, with a focus on identifying potential risk factors for cognitive decline. According to a 2020 report of the Lancet Commission, 40% of dementia cases are associated with 12 risk factors, including lifestyle factors such a smoking, alcohol consumption, physical inactivity and obesity, while the role of diet is less clear [3]. While abundant evidence from observational research suggests that adherence to healthy dietary patterns—such as the Mediterranean, Dietary Approaches to Stop Hypertension (DASH), and Mediterranean-DASH Intervention for Neurodegenerative Delay (MIND) diets, which prioritize plant-based over animal-based foods, may help to slow down cognitive decline in older adults, evidence from randomized controlled trials is limited [4–6].

In addition to dietary patterns, research has also explored the roles of specific macronutrients in cognitive health [7]. Protein, in particular, has gained attention for its potential cognitive benefits, possibly due to specific amino acids [7–10]. However, findings from epidemiological studies in older adults remain inconsistent. Several cross-sectional [11–13] and longitudinal studies [14–17] have observed a positive association between higher protein intake and better cognitive function or slower cognitive decline, while other cross-sectional [18–20] and longitudinal studies [21, 22] found no statistically significant association. One study reported positive associations only in older females [23]. In contrast, a cross-sectional study found higher protein intake in older adults with dementia in French nursing homes [24]. Finally, randomized controlled trials showed no significant cognitive benefits from protein supplementation [25–27], although the 12–24 week follow-up may have been too short to show effects.

Variation in the source and quality of protein intake between studies may partly explain inconsistent findings. Animal-based proteins are often considered of higher quality due to their complete amino acid profile [28], which may better support cognitive health through specific amino acids [8–10]. However, plant-based diets, linked to broader health benefits [29], have also been associated with better cognitive function [30, 31], although results are not unequivocal [32]. Studies comparing animal- and plant-based protein sources show inconsistent results [11, 15, 17].

Given the current shift from animal to plant-based proteins to promote sustainable diets [33], and the limited research directly comparing animal- and plant-based protein sources in relation to cognitive function, along with the inconsistent findings from previous studies on the association between protein intake and cognition, further investigation is warranted. Therefore, this study aims to investigate the association between total, animal-based, and plant-based protein intake and cognitive function and decline in community-dwelling older adults participating in the Longitudinal Aging Study Amsterdam (LASA).

## Methods

### Study design

This study used data from the Longitudinal Aging Study Amsterdam (LASA), a cohort study that collects information from a representative sample of the Dutch population aged 55 and older across three regions in the Netherlands: Amsterdam, Zwolle, and Oss. Details of the LASA study are described elsewhere [34, 35]. In short, participants were recruited using population registers from these areas. Initiated in 1992, LASA includes three cohorts and several waves of data collection, with follow-ups approximately every three years. The first cohort began in 1992/1993 (participants aged 55–85 at baseline), followed by the second cohort in 2002/2003 and the third in 2012/2013 (participants aged 55–65 at baseline). Data were gathered through face-to-face interviews at participants’ homes, encompassing a main interview, a medical interview, and a self-administered questionnaire. The data collection covers a wide range of topics related to the physical, cognitive, emotional, and social aspects of ageing. For the present study, we also included data from the ancillary Nutrition and Food-related Behavior Study conducted in 2014–2015, which involved a subset of 1,439 LASA participants and collected information on dietary intake with a 238-item Food Frequency Questionnaire (FFQ) at a single time point [36]. For the present study, cognitive and other measurements from the previous LASA cycle (2011–2013) served as the baseline (T0), followed by assessments in 2015/2016 (T1), 2018/2019 (T2), and 2021/2022 (T3). The LASA study received ethical approval from the Medical Ethics Committee of VU University Medical Center, and all participants provided written informed consent prior to participation.

### Study sample

**Fig. 1:**
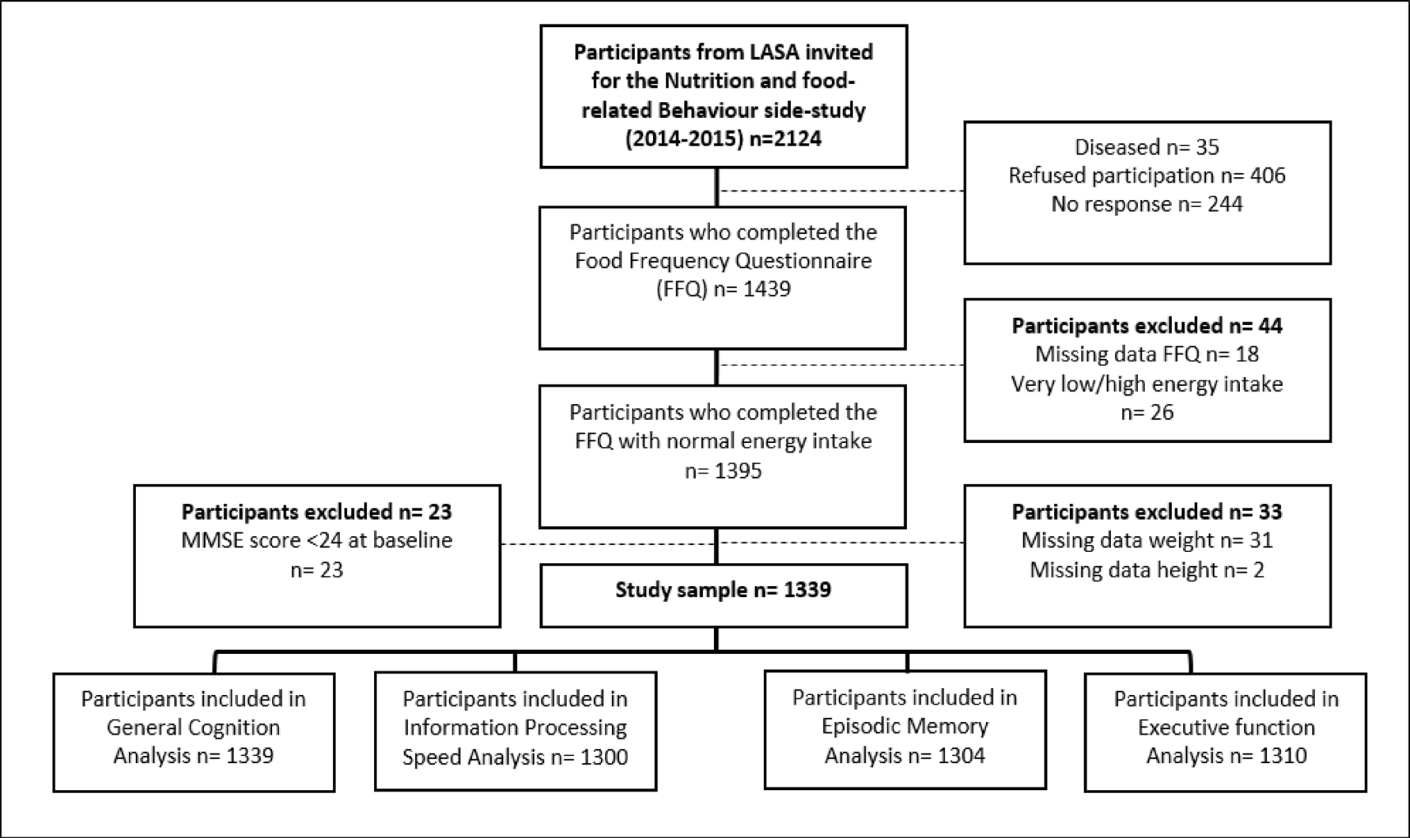
Flowchart of the study sample of Dutch community-dwelling older adults aged 55 and older from the Longitudinal Aging Study Amsterdam

The study sample consisted of Dutch older men and women aged 55 and older who participated in the Nutrition and Food-related Behavior ancillary study of LASA and completed the FFQ (*n* = 1439) [36]. To ensure data reliability, participants were excluded if they had ≥ 10 missing items on the FFQ (n = 18) or reported extremely low or high energy intakes (n = 26; < 800 kcal/d or > 4,000 kcal/d for males and < 500 kcal/d or > 3,500 kcal/d for females) [37]. Those with missing baseline weight and height data were also excluded (n = 33), as these measures were needed to calculate total protein intake in grams per kilogram of adjusted body weight per day (g/kg adjusted body weight (BW)/d). Additionally, participants with a very poor baseline cognitive status (MMSE < 24) were excluded (n = 23) to improve data accuracy by reducing potential FFQ misunderstandings [38]. After applying these inclusion and exclusion criteria, the final sample consisted of 1,339 participants aged 55 + (median age 65.2 y, IQR 61.0;72.1), though the sample size varied across cognitive tests due to additional missing data per test; participants required at least one cognitive function measurement (Fig. 1).

### Measurements

#### Dietary protein intake

Dietary protein intake was assessed at baseline using a validated self-administered FFQ developed for the Healthy Life in an Urban Setting (HELIUS) study, with 76 questions on 238 food items [39, 40]. The HELIUS FFQ’s relative validity was evaluated in a subsample of 88 older participants (mean age: 71.9 years) who additionally completed three 24-h dietary recalls [39]. For energy and macronutrient intakes, the average bias at the group level was within 5%, with Pearson correlation coefficients ranging from 0.26 to 0.72 and moderate to high agreement across quintiles. Specifically for protein intake, Pearson correlations were 0.39 for total protein, 0.49 for plant-based protein intake, and 0.41 for animal-based protein intake [39]. These validity estimates are comparable to those typically observed for FFQs and are considered sufficient for use in dietary intake research [41]. Participants reported consumption frequency and portion sizes of the products over the previous four weeks. Portion sizes were described using kitchen utensils, such as a bowl or spoon, and images. Dietary protein intake was calculated by linking food items to the Dutch Food Composition Database (2011) [42]. Dietary protein intake was categorized into animal-based sources (meat, fish, dairy, and eggs) and plant-based sources (grains, legumes, nuts, seeds, and vegetables), measured in grams per day. Total, animal-based, and plant-based protein intakes were expressed as percentages of total energy intake (% energy). Total protein intake was also expressed in g/kg adjusted BW/d) by using the closest weight that would categorize the individual within a healthy body mass index (BMI) range of 18.5 to 25.0 kg/m^2^ for adults younger than 71 years of age and 22.0 to 27.0 kg/m^2^ for adults aged 71 years and older.

#### Cognitive function

Cognitive function was assessed by trained interviewers in participants’ homes during the medical interview at four time points (T0-T3). Global cognition was evaluated using the Mini-Mental State Examination (MMSE) [43], and domain-specific cognition was measured with the following tests: the Coding Task for information processing speed, the 15 Words Test (15WT) for episodic memory [44], and the Word Fluency Test for executive functioning [45].

The MMSE, which screens global cognition in older adults [43] assesses five domains: orientation, immediate memory, attention and concentration, delayed recall, and language [46]. Scores range from 0 to 30, with higher scores indicating better global cognitive function.

Information processing speed was assessed with an adapted Alphabet Coding Task-15, a letter substitution task [47]. Participants verbally matched characters from two rows in a one-minute trial, repeated three times. This coding task differed from the original by requiring verbal instead of written responses because writing may slow down the processing speed, and written responses were difficult to read during analyses in a previous pilot study [48]. The average score, ranging from 2.0 to 49.3, was used in analysis, with higher scores indicating higher processing speed.

Episodic memory was measured by the 15WT, an adapted version of the Auditory Verbal Learning Task, where participants recalled as many of 15 words as possible over three trials and after a 20-min delay [44]. The test used in LASA consists of three trials instead of five because of limited interview time. After participants recalled as many words as possible per trial (range 0–15), they had to name the words again after 20 min to measure the delayed recall (range 0–15). Two different word lists were used to reduce practice effects. For the analyses, immediate recall scores (range 0–45), maximum recall (range 0–15), and delayed recall (range 0–15) were combined (average scale range 0–25), with higher scores indicating better memory.

Executive function was measured using a verbal fluency test [45, 49] in which participants named words beginning with the letter D and listed animals in two one-minute tasks. Repeated words counted only once, with scores ranging from 0 to 36 for letter D words and 0 to 40 for animals. The mean score across tasks (scale range 0–38) was used in the analyses, with higher scores indicating better executive function.

All cognitive test scores were standardized as z-scores based on the mean (M) and standard deviation (SD) at baseline, allowing comparison across cognitive domains. For cognitive tests with multiple scores, the z-scores were combined into domain scores based on previous studies [50, 51]: Global cognition = Z_MMSE_, Information processing speed = Z_Coding Task Mean_, Episodic memory = (Z_15WT Total_ + Z_15WT Maximal_ + Z_15WT Delayed Recall_) / 3, Executive function = (Z_Verbal Fluency Animal_ + Z_Verbal Fluency Letter D_) / 2.

#### Covariates

During the main interview of the regular LASA wave, data were collected on: sex at birth (male/female), age (in years), the highest level of education completed by the participant (categorized as low for not completed and completed elementary education; middle for lower vocational, general intermediate, intermediate vocational, and general secondary education; and high for higher vocational education and above), and living situation (categorized as living alone or not). In addition, data were collected on the self-reported number of chronic diseases, scored on a scale from 0 to 7 (including non-specific chronic lung disease, heart disease, peripheral arterial disease, stroke, diabetes mellitus, arthritis, and malignancies) (categorized as none, 1 or 2, or > 2 chronic diseases). Depressive symptoms were assessed using the Center for Epidemiologic Studies Depression Scale (CES-D), a 20-item self-report scale with a total score ranging from 0 to 60 (higher scores indicate more depressive symptoms) [52]. Physical activity in the past two weeks was assessed using the validated LASA Physical Activity Questionnaire (by multiplying the frequency, duration, and Metabolic Equivalent of Task scores (METs) for all activities), resulting in a MET hours/week score [53, 54]. During the medical interview of the regular LASA wave, data were collected on self-reported smoking status (categorized as never, former, or current smoker) and the weekly number of alcohol consumption (categorized as none, light, moderate, or (very) excessive consumption) depending on the number of days and amount of alcohol consumed [55]. BMI information was obtained by dividing body weight (measured to the nearest 0.1 kg on a calibrated scale) by height squared (measured to the nearest 0.001 m with a stadiometer) (categorized as follows: for those ≤ 70 years, underweight (BMI < 18.5 kg/m^2^), healthy weight (BMI 18.5–24.9 kg/m^2^), overweight (BMI 25.0–29.9), and obese (BMI > 30.0); for those > 70 years, underweight (BMI < 22.0 kg/m^2^), healthy weight (BMI 22.0–28.0 kg/m^2^), overweight (BMI 28.0–30.0 kg/m^2^), and obese (BMI > 30 kg/m^2^). If weight or height data were missing at baseline, self-reported baseline values or values obtained during the Nutrition and Food-related Behaviour side Study were used [36]. Total energy intake (kcal/d) was assessed during the Nutrition and Food-related Behaviour side Study [36] using the FFQ, based on the Dutch Food Consumption Table (2011) [42]. Diet quality was assessed with the Dutch Healthy Diet Index (DHD-15) based on the FFQ data. This index, evaluating adherence to the Dutch dietary guidelines [56], rated 13 (vegetables, fruits, whole grain products, legumes, nuts and seeds, dairy, fish, tea, fats and oils, red meet, processed meet, sugar-sweetened bevarages and fruit juices, alcohol) instead of 15 components on a scale of 0–10 due to the lack of data on salt and type of coffee consumption, resulting in a total score ranging from 0 (no adherence) to 130 (complete adherence) [56].

#### Statistical analyses

Baseline (T0) characteristics of the study sample were reported, for the total sample and stratified by sex, as means (M) and standard deviations (SD) for normally distributed continuous variables, medians and interquartile ranges (IQR) for non-normally distributed variables, and frequencies with percentages for categorical variables.

For the main analyses, associations between quartiles (Q) of protein intake % energy (total, animal-, and plant-based) and cognitive test scores (z-scores) and cognitive decline with age were examined. We categorized protein intake (% energy) into quartiles, which is a common practice in nutritional epidemiology to avoid model misspecification and reduce the influence of outliers [57]. Quartiles were defined as Q1 (< 25th percentile (< 13.9), reference), Q2 (25-50th percentile (13.9–15.5)), Q3 (50-75th percentile (15.5–17.1)), and Q4 (> 75th percentile (> 17)). Linear mixed models in STATA 18 were chosen to handle missing data across time points and account for individual differences in cognitive scores and change over time, with random intercepts for individuals and, if necessary, a random slope for age (as time-dependent variable) and an unstructured covariance matrix. The inclusion of the random slope was determined by comparing model log-likelihoods. Normality assumptions for residuals were assessed through histograms and Q-Q plots, with all cognitive z-scores showing normality in regression residuals. Following the approach of Nooyens et al. [58] two types of models were used. The first model, referred to as the "level model," assessed the association between quartiles of protein intake and cognitive function level. The second model, referred to as the "change model", examined whether cognitive decline with age differed by quartiles of protein intake by including an interaction term between age and protein intake.

*Protein intake & level of cognitive function.* Three models were used to evaluate associations between protein intake and level of cognitive function during aging. The first model (Model 1) adjusted for age (years) and age^2^ as time-dependent variables, as well as level of education, considering their impact on cognitive function [59, 60]. Age was modeled quadratically because of the known non-linear relationship between age and cognitive function [61]. In addition, the model was additionally adjusted for total energy intake following the multivariate nutrient density model [62]. Interaction between sex and protein was tested in the crude model, with results stratified by sex if p < 0.05, as previous research has shown different associations of protein intake with cognitive function between males and females [23]. The second model (Model 2) additionally adjusted for sex (if no interaction), living status, chronic diseases, depressive symptoms, physical activity, smoking status, alcohol consumption, and BMI (baseline measurements, T0). These potential confounders align with those selected in previous studies investigating the association between protein intake and cognitive function and decline [11, 15–17, 22]. Animal protein models were adjusted for plant protein and vice versa. The third model (Model 3) further adjusted for diet quality (DHD-15 index) to separate associations of (plant/animal-based) protein intake from overall diet quality, which is also linked to cognitive function [63, 64]. Positive associations indicate higher cognitive function in higher protein intake quartiles (Q2-Q4) compared to Q1.

*Protein intake & cognitive decline.* To analyze the association between protein intake and cognitive decline with age, interaction terms between protein intake quartiles and age were added to the three models described above. Statistically significant positive protein x age interactions (p ≤ 0.05) indicate slower cognitive decline with ageing for higher protein intake quartiles (compared to Q1). Sex was tested as an effect modifier in the crude model by adding a three-way interaction term of sex x protein x age with sex-stratified results if p < 0.05. Decline models included the same confounders as the level models.

Associations between protein intake, cognitive function, and cognitive decline were presented in tables (β, 95% CI) and graphs, with plots for the lowest (Q1) and highest (Q4) quartiles to illustrate differences in cognitive levels and decline. For the plots, age and age^2^ were centered at 55 years. In cases where Q2 or Q3 showed statistically significant protein x age interactions but not Q4, Q2 and Q3 were also plotted. Sex-stratified plots were provided when interaction with sex was statistically significant.

Sensitivity analyses explored protein intake in g/kg adjusted BW/day, divided into four quartiles for the fully adjusted models (Model 3).

## Results

### Population characteristics

Baseline characteristics of participants (total and stratified by sex) are shown in Table 1. The final study sample consisted of 1339 participants (52.4% female) with a median age at baseline of 65.2 years (IQR = 61.0–72.1). Female were on average lower educated, more likely to live alone, had more depressive symptoms (CES-D), were more physically active (MET-hours/week) and had a higher diet quality (DHD-15) than males (Table 1). Mean protein intake of males and female was almost identical, with a combined mean intake of 15.6 ± 2.6 (% energy), 1.1 ± 0.3 (g/kg adjusted BW/d), 9.4 ± 2.7 (% energy) for animal-based protein, and 6.2 ± 1.3 (% energy) for plant-based protein (mean ± SD). Median MMSE score at baseline was 29.0 (IQR = 28.0,30.0) and was similar between males and females. Participants were followed up for a median of 9.2 years (IQR = 8.9–9.9).

**Table 1:** Baseline characteristics of the study sample of Dutch community-dwelling older adults of the Longitudinal Aging Study Amsterdam (LASA)

Characteristic	Total	Males	Females
Number of subjects Age (years), median (IQR)	1339 65.2 (61.0;72.1)	637 65.3 (61.4;71.9)	702 65.2 (60.8;72.4)
*Education, n (%)*			
Low	161 (12.0)	61 (9.6)	100 (14.2)
Middle	780 (58.3)	333 (52.3)	447 (63.7)
High	398 (29.7)	243 (38.1)	155 (22.1)
*Living situation, n (%)*			
Living alone	338 (25.2)	96 (15.1)	242 (34.5)
Living with partner	1001 (74.8)	541 (84.9)	460 (65.5)
*Number of chronic diseases, n (%)*			
None	407 (30.4)	220 (34.5)	187 (26.6)
One	532 (39.7)	239 (37.5)	293 (41.7)
Two or more	400 (29.9)	178 (27.9)	222 (31.6)
Depressive symptoms sum (CES-D score)^a^, median (IQR)	5.0 (2;10)	4.0 (1.0;8.0)	7.0 (3.0;11.0)
Physical activity MET-hours/week^a^, median (IQR)	53.5 (33.0;79.4)	43.0 (26.3;69.0)	62.8 (41.8;85.5)
Diet quality (DHD-15, range 0–130), mean ± SD	82.4 ± 16.1	78.5 ± 16.0	85.9 ± 15.3
*Smoking status, n (%)*			
Current smoker	155 (11.6)	75 (11.8)	80 (11.4)
Former smoker	794 (59.3)	427 (67.0)	367 (52.3)
Never smoker	371 (27.7)	127 (19.9)	244 (34.8)
Missing	19 (1.4)	8 (1.3)	11 (1.6)
*Alcohol use, n (%)*			
Non-drinker	162 (12.1)	54 (8.5)	108 (15.4)
Light drinker	624 (46.6)	246 (38.6)	378 (53.8)
Moderate drinker	455 (34.0)	263 (41.3)	192 (27.4)
(very) Excessive drinker	79 (5.9)	68 (10.7)	11 (1.6)
Missing	19 (1.4)	6 (0.9)	13 (1.9)
*BMI*^*b*^*, n (%)*			
Underweight	14 (1.0)	9 (1.4)	5 (0.7)
Normal weight	578 (43.2)	247 (38.8)	331 (47.2)
Overweight	453 (33.8)	245 (38.5)	208 (29.6)
Obese	294 (22.0)	136 (21.4)	158 (22.5)
*Dietary intake, mean* ± *SD*			
Energy (Kcal/d)	2083.9 ± 574.3	2313.5 ± 589.8	1875.5 ± 471.8
Total protein (% energy)	15.6 ± 2.6	15.4 ± 2.5	15.8 ± 2.7
Total Protein (g/kg adjusted BW/d)	1.1 ± 0.3	1.1 ± 0.3	1.1 ± 0.3
Total Protein (g/d)	80.6 ± 23.5	83.4 ± 24.8	73.6 ± 19.8
Animal protein (% energy)	9.4 ± 2.7	9.3 ± 2.6	9.6 ± 2.8
Plant protein (% energy)	6.2 ± 1.3	6.1 ± 1.3	6.3 ± 1.4
*Cognitive function*^*a*^			
MMSE, median (IQR)	29.0 (28.0;30.0)	29.0 (28.0;29.0)	29.0 (28.0;30.0)
Coding task, mean ± SD	29.4 ± 6.2	28.5 ± 5.9	30.2 ± 6.4
15WT^c^, mean ± SD	14.3 ± 3.5	13.4 ± 3.5	15.2 ± 3.3
Verbal fluency^d^, mean ± SD	16.9 ± 4.4	16.9 ± 4.6	17.0 ± 4.3

BMI, body mass index; BW, body weight; CES-D, Center for Epidemiologic Studies Depression scale; IQR, interquartile range; MET, metabolic equivalent of task; MMSE, Mini-mental state examination; SD, standard deviation; 15WT, 15-word test. Values are displayed as mean (M) ± standard deviations (SD) or median and interquartile range (IQR), and frequencies are presented as n (%). ^a^Variables with missing data (≤ 5.0%)

^b^ BMI was categorised as follows: for those ≤ 70 years, underweight (BMI < 18.5 kg/m^2^), healthy weight (BMI 18.5–24.9 kg/m^2^), overweight (BMI 25.0–29.9 kg/m^2^), and obese (BMI > 30.0 kg/m^2^); for those > 70 years, underweight (BMI < 22.0 kg/m^2^), healthy weight (BMI 22.0–28.0 kg/m^2^), overweight (BMI 28.0–30.0 kg/m^2^), and obese (BMI > 30 kg/m^2^). ^c^ Mean score of total, maximal and delayed 15WT. ^d^ Mean score of correct D-words and animal-words

### Total protein intake and level of cognitive function

**Fig. 2:**
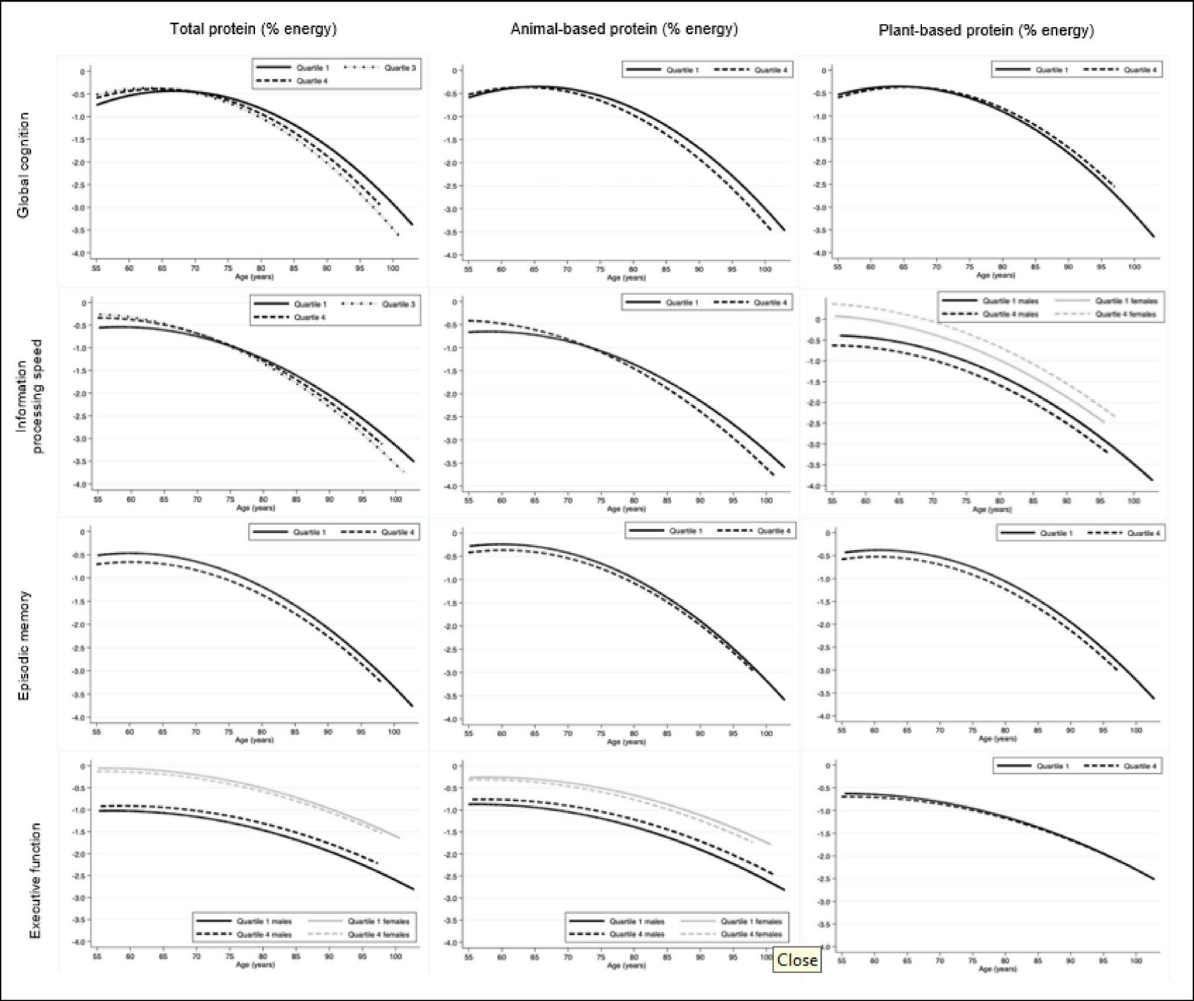
Association between total, animal-based and plant-based protein intake and cognitive function and decline with ageing in older adults of the Longitudinal Aging Study Amsterdam (LASA)

Table 2 presents the linear mixed model results for the association between protein intake (% energy) and level of cognitive function. Figure 2 visualizes cognitive function differences between Q1 and Q4. The analysis of protein intake and executive function was stratified by sex due to a statistically significant protein x sex interaction in Q4 versus Q1 (p = 0.02) in the crude model but stratified (adjusted) associations were not statistically significant. Compared to the lowest quartile, participants in higher protein intake quartiles had lower episodic memory scores (Q2 vs. Q1: β = − 0.24, 95%CI: − 0.36 to − 0.12; Q3 vs. Q1: β = − 0.17, 95%CI: − 0.29 to − 0.06; Q4 vs. Q1: β = − 0.18, 95%CI: − 0.30 to − 0.06). No statistically significant associations were observed for the other cognitive tests.

**Table 2:** Association between total protein intake (% energy) and level of cognitive function^a^ in Dutch older adults of the Longitudinal Aging Study Amsterdam (LASA)

		Model 1^b^ β (95%CI)	Model 2^c^ β (95%CI)	Model 3^d^ β (95%CI)
Global cognition	Quartile 2	− 0.0 (− 0.13,0.13)	0.00 (− 0.12,0.14)	− 0.01 (− 0.14,0.12)
	Quartile 3	0.00 (− 0.12,0.13)	0.00 (− 0.12,0.14)	− 0.01 (− 0.13,0.12)
	Quartile 4	0.00 (− 0.13,0.13)	0.02 (− 0.11,0.15)	0.01 (− 0.13,0.14)
Information processing speed	Quartile 2	− 0.08 (− 0.22,0.05)	− 0.09 (− 0.22,0.05)	− 0.09 (− 0.22,0.05)
	Quartile 3	0.06 (− 0.07,0.20)	0.08 (− 0.05,0.21)	0.08 (− 0.05,0.22)
	Quartile 4	0.06 (− 0.08,0.20)	0.07 (− 0.07,0.21)	0.07 (− 0.07,0.21)
Episodic memory	Quartile 2	− 0.22 (− 0.34,− 0.10)***	− 0.22 (− 0.34,− 0.10)***	− 0.24 (− 0.36,− 0.12)***
	Quartile 3	− 0.18 (− 0.30,− 0.05)**	− 0.16 (− 0.28,− 0.04)**	− 0.17 (− 0.29,− 0.06)**
	Quartile 4	− 0.17 (− 0.30,− 0.04)**	− 0.16 (− 0.28,− 0.04)*	− 0.18 (− 0.30,− 0.06)**
Executive function *Males*	Quartile 2	0.08 (− 0.07,0.23)	0.08 (− 0.07,0.24)	0.06 (− 0.09,0.21)
	Quartile 3	0.08 (− 0.08,0.23)	0.10 (− 0.06,0.25)	0.09 (− 0.07,0.24)
	Quartile 4	0.12 (− 0.05,0.28)	0.15 (− 0.02,0.32)	0.14 (− 0.03,0.30)
*Females*	Quartile 2 Quartile 3 Quartile 4	− 0.10 (− 0.24,0.05) − 0.05 (− 0.19,0.10) **− **0.09 (− 0.23,0.05)	− 0.07 (− 0.21,0.07) − 0.02 (− 0.17,0.12) − 0.06 (− 0.20,0.08)	− 0.08 (− 0.22,0.07) − 0.03 (− 0.17,0.12) − 0.07 (− 0.21,0.07)

^a^Cognitive function level (z-score) differences are presented as unstandardized regression coefficients β (95% CI) for protein intake quartiles Q2, Q3, and Q4, compared to the reference quartile, Q1. Protein intake quartiles (% energy) are defined as: Q1 < 13.9; Q2 13.9–15.5; Q3 15.5–17.1; Q4 > 17. Negative β values indicate lower cognitive function levels relative to Q1. Results were stratified by sex in case there was significant interaction by sex (p ≤ 0.05) in model 1. Sample sizes were 1339 for global cognition, 1300 for information processing speed, 1304 for episodic memory, and 1310 for executive function. Estimates in bold are statistically significant * p ≤ 0.05 **p ≤ 0.01 ***p ≤ 0.001. ^b^Model 1: adjusted for age, age2, educational level, and energy intake. ^c^ Model 2: additionally adjusted for sex (if no interaction), living status, number of chronic diseases, depressive symptoms, physical activity, smoking status, alcohol use, and Body Mass Index. ^d^ Model 3: additionally adjusted for diet quality (DHD-15, range 0–130)

### Total protein intake and cognitive decline with ageing

Table 3 and Fig. 2 present the association between protein intake (% energy) and cognitive decline with age, incorporating the interaction terms for protein x age. No statistically significant effect modification by sex was observed (*p* ≥ 0.05), so results were not stratified by sex. Statistically significant interactions were observed between protein intake (% energy) and age for the association with global cognition and information processing speed but not for episodic memory and executive function. For global cognition, compared to the lowest quartile, participants in the third quartile of protein intake showed a faster decline with age compared to the lowest quartile (protein x age: Q3 versus Q1: β = − 0.02, 95%CI: − 0.03 to − 0.00). Similarly, for information processing speed, the third quartile of protein intake was associated with a faster decline compared to the lowest quartile (protein x age: Q3 versus Q1: β = − 0.02, 95%CI: − 0.03 to − 0.01).

**Table 3:** Association between total protein intake (% energy) and cognitive decline^a^ with age in Dutch older adults of the Longitudinal Aging Study Amsterdam (LASA)

		Model 1^b^ β (95%CI)	Model 2^c^ β (95%CI)	Model 3^d^ β (95%CI)
Global cognition	Quartile 2 × age	− 0.01 (− 0.10,− 0.02)	− 0.01 (− 0.03,0.00)	− 0.01 (− 0.03,0.00)
	Quartile 3 × age	− 0.02 (− 0.11,− 0.03)**	− 0.02 (− 0.03,− 0.00)*	− 0.02 (− 0.03,− 0.00)*
	Quartile 4 × age	− 0.01 (− 0.03,0.00)	− 0.01 (− 0.03,0.00)	− 0.01 (− 0.03,0.00)
Information processing speed	Quartile 2 × age	− 0.01 (− 0.02,0.00)	− 0.01 (− 0.02,0.00)	− 0.01 (− 0.02,0.00)
	Quartile 3 × age	− 0.02 (− 0.03,− 0.01)*	− 0.02 (− 0.03,− 0.01)**	− 0.02 (− 0.03,− 0.01)**
	Quartile 4 × age	− 0.01 (− 0.02,0.00)	− 0.01 (− 0.02,0.00)	− 0.01 (− 0.02,0.00)
Episodic memory	Quartile 2 × age	− 0.01 (− 0.02,0.00)	− 0.01 (− 0.02,0.01)	− 0.01 (− 0.02,0.01)
	Quartile 3 × age	− 0.00 (− 0.01,0.01)	0.00 (− 0.01,0.01)	0.00 (− 0.01,0.01)
	Quartile 4 × age	0.00 (− 0.01,0.01)	0.00 (− 0.01,0.01)	0.00 (− 0.01,0.01)
Executive function	Quartile 2 × age	0.00 (− 0.01,0.01)	0.00 (− 0.01,0.01)	0.00 (− 0.01,0.01)
	Quartile 3 × age	− 0.00 (− 0.01,0.01)	− 0.00 (− 0.01,0.01)	− 0.00 (− 0.01,0.01)
	Quartile 4 × age	0.00 (− 0.01,0.01)	0.00 (− 0.01,0.01)	0.00 (− 0.01,0.01)

^a^Cognitive function decline (z-score) differences are presented as unstandardized regression coefficients β (95% CI) for protein intake quartiles Q2, Q3, and Q4, compared to the reference quartile, Q1. Protein intake quartiles (% energy) are defined as: Q1 < 13.9; Q2 13.9–15.5; Q3 15.5–17.1; Q4 > 17. Models test decline differences using interaction terms for protein x age. Positive β values for the protein x age interaction indicate slower decline with age relative to Q1. Sample sizes were 1339 for global cognition, 1300 for information processing speed, 1304 for episodic memory, and 1310 for executive function. Estimates in bold are statistically significant * p ≤ 0.05 **p ≤ 0.01 ***p ≤ 0.001. ^b^ Model 1: adjusted for age, age2, educational level, and energy intake. ^c^ Model 2: additionally adjusted for sex, living status, number of chronic diseases, depressive symptoms, physical activity, smoking status, alcohol use, and Body Mass Index. ^d^ Model 3: additionally adjusted for diet quality (DHD-15, range 0–130)

### Source of protein and level of cognitive function

Supplementary Table 1 and Fig. 2 show the associations between protein intake by source (animal-based or plant-based, % energy) and levels of cognitive function. Due to a statistically significant protein x sex interaction in Q4 versus Q1 (p = 0.01), the association between animal-based protein intake and executive function was stratified by sex, but adjusted results showed no statistically significant associations. In model 1, animal-based protein intake showed significant negative associations with executive function in females in Q3 and Q4 versus Q1, but these associations were not observed in the more fully adjusted models (model 2 and 3). For animal-based protein intake, no statistically significant associations were observed with any cognitive domains after adjustment for confounders. For plant-based protein, the association with information processing speed was stratified by sex because of statistically significant interaction in Q4 versus Q1 (p = 0.04). For plant-based protein intake, females in the highest quartile showed higher levels of information processing speed compared to the lowest quartile (Q4 versus Q1: β = 0.31, 95%CI: 0.09 to 0.53) while no association was observed in males. For episodic memory, results across the three models were inconsistent. No significant associations were observed for models 1 and two, while after adjustment for diet quality in model 3, participants in the highest quartile of plant-based protein intake showed lower levels of episodic memory compared to the lowest quartile (Q4 versus Q1: β = − 0.16, 95%CI: − 0.30 to − 0.02). No associations were observed for global cognition or executive function.

### Source of protein and cognitive decline with ageing

Supplementary Table 2 and Fig. 2 show the associations between protein intake source (animal-based or plant-based, % energy) and cognitive decline with age. No statistically significant interactions were found between animal-based protein (% energy) and age, except for information processing speed. Participants in the third and fourth quartiles of animal-based protein intake showed a faster decline in processing speed compared to the lowest quartile (Q3 versus Q1: β = − 0.02, 95%CI: − 0.03 to − 0.01; Q4 versus Q1: β = − 0.01, 95%CI: − 0.02 to − 0.00). No statistically significant interactions were found between plant-based protein (% energy) and age in any of the cognitive domains.

Figure 2* shows the changes of cognitive function as a function of calendar age for the lowest protein intake quartile (quartile 1) and the highest protein intake quartile (quartile 4) (Q2 or Q3 were only plotted when the interaction term age x protein intake was statistically significant for Q2 or Q3 and not for Q4). Graphs were stratified by sex if there was interaction by sex in the level of cognitive function models (p* ≤ *0.05). Models include interaction terms of protein x age. Associations were adjusted for age (centered at 55 years), age*^*2*^*, educational level, sex (if no interaction), energy intake (kcal), plant protein (% energy) (for animal protein model), animal protein (% energy) (for plant protein model), living status, chronic diseases, depressive symptoms, physical activity, smoking status, alcohol use, Body Mass Index, and diet quality (DHD-15,* range 0–130*)*.

### Sensitivity analyses

Sensitivity analyses based on g/kg adjusted BW/d showed associations that are for the most part in line with our main analyses. Participants in the highest quartile of protein intake (Q4) showed lower levels of episodic memory and a faster decline in global cognition compared to the lowest quartile, consistent with our main analyses. Contrasting our main analyses, no association was observed with decline in processing speed and participants in the highest quartile (Q4) showed less decline in episodic memory compered to the lowest quartile (Q1). (Supplementary Tables 3 and 4).

## Discussion

This longitudinal study of older adults (55 + years) found that higher total protein intake was statistically significantly associated with lower episodic memory and faster decline in global cognition and processing speed. Higher animal-based protein intake was associated with faster decline in processing speed, whereas higher plant-based protein intake was associated with higher processing speed in females and lower episodic memory. No associations were found for executive functioning.

Our findings partially align with previous studies reporting no significant associations between protein intake and cognitive outcomes [18–22], as most associations in our study were also not statistically significant. Our results are, however, in contrast with previous cross-sectional and longitudinal studies that generally show a positive relationship between total protein intake and cognitive function in older adults [11–16, 23]. Our negative associations observed between higher total protein intake and cognitive function are consistent with a study conducted in French nursing homes, where higher total protein intake was observed among older adults with dementia compared to those without dementia [24].

In our study, higher animal-based protein intake was associated with a faster decline in processing speed, while higher plant-based protein intake was linked to improved processing speed in females but lower episodic memory. These results are not in line with a previous cross-sectional study that found a positive association between animal-based protein intake and greater information processing speed in older adults, while no association was found for memory function or executive function, nor were these cognitive tests associated with plant-based protein intake [11]. Additionally, also contrasting our findings, a previous longitudinal study observed slower decline in cognitive function with higher animal-based protein intake and a faster decline with higher plant-based protein intake [17]. Finally, another longitudinal study observed that total, animal-based and plant-based protein intake were associated with a slower cognitive decline, with stronger associations for plant-based protein intake [15]. Results on the association between protein sources and cognitive function thus remains inconsistent.

Differences in study findings may be attributed to variations in study populations, sample sizes, or the specific cognitive domains tested. Geographical factors may also play a role, given that primary protein sources differ between regions and countries and these sources differ in nutrient composition [65, 66]. In the Netherlands, meat and poultry (23%) and dairy (26%) are the dominant protein sources [65], all of animal origin, potentially influencing associations observed. The mechanisms underlying the associations observed in this study remain unclear. Although protein and specific amino acids have been suggested to support cognitive health by providing precursors for neurotransmitters such as dopamine (from tyrosine) and serotonin (from tryptophan), as well as indirectly through maintaining muscle mass and function [8–10], the more favourable association we observed for plant- versus animal-based proteins with processing speed suggests that cognitive effects may relate more to the overall nutritional profile of protein sources than to protein quality itself. For example, animal-based protein sources often co-occur with saturated fats and other nutrients linked to poorer vascular health [67], which is known to negatively impact cognition [68]. Conversely, plant-based protein sources (e.g., legumes, nuts, and whole grains), are low in saturated fats and rich in anti-inflammatory and antioxidant compounds which may benefit cognitive health [69], though potential deficiencies in critical nutrients such as vitamin B12, long-chain omega-3 fatty acids and iron should be considered [69]. As current evidence on the role of plant versus animal based protein sources in cognitive health remains limited and contradictory, [11, 15, 17] further research is warranted.

From a practical perspective, “higher protein intake” in our study refers to intakes above the first quartile, that is, ≥ 13.6% of total energy or ≥ 0.9 g/kg adjusted BW/day. This aligns with the recommended intake of at least 0.83 g/kg/d [70, 71], or 1.0 g/kg/d[72] for older adults. The highest quartile reached ≥ 17% of energy or ≥ 1.3 g/kg adjusted BW/day, levels that have been suggested in some studies to help maintain physical function in old age [73]. Adequate protein intake remains essential for muscle and functional health. However, among older adults with relatively high protein intake in the general population, higher intake, especially from animal sources, may not benefit cognition and could relate to faster decline in some domains. Shifting towards plant-based proteins, while ensuring sufficient protein intake, may better support cognitive and overall health in older adults.

This study has several strengths. It includes a large sample of older adults from a population-based cohort and adjusts for many potential confounding factors. The longitudinal design, with repeated cognitive tests across different domains, allows us to examine changes within individuals over time and improves the statistical power. Using multiple cognitive tests increases sensitivity to subtle changes, giving a detailed and accurate picture of cognitive function. We also assessed total protein intake in two ways: as a percentage of energy intake (using the multivariate nutrient density model) and as g/kg adjusted BW/d in a sensitivity analysis. Finally, by separating animal- and plant-based protein intake, we could explore more precisely how different protein sources relate to cognitive outcomes.

In addition to the study’s strengths, certain limitations must be addressed. Firstly, the use of a Food Frequency Questionnaire (FFQ) is subject to recall bias, which may introduce inaccuracies in reporting dietary habits [74]. This could lead to non-differential misclassification and an underestimation of observed associations. However, previous research has shown that the FFQ has acceptable to good relative validity for assessing dietary intake in Dutch older adults [39]. Additionally, the FFQ was administered only once, which assumes constant protein intake over time and may not account for actual dietary fluctuations. Although previous studies have observed moderate stability of protein intake over time [75], individual dietary changes do occur and may lead to non-differential misclassification and attenuation of associations. Lastly, our statistical significant findings should be interpreted with caution, as they may be chance results due to the large number of statistical tests performed, increasing the risk of type I error.

In conclusion, higher overall protein intake was associated with lower episodic memory and faster decline in global cognition and processing speed. Animal-based protein was linked to faster decline in processing speed, while plant-based protein was associated with better processing speed in females but lower episodic memory in the total sample. Given the inconsistent findings across studies, definitive conclusions remain difficult. However, our contrasting results for plant- versus animal-based proteins and processing speed suggest that that cognitive outcomes may relate more to the nutrient content of protein-rich foods than to protein quality. These findings support current recommendations to shift toward more plant-based protein sources [76].

## Supplementary Information

Below is the link to the electronic supplementary material.

Supplementary file1

## Data Availability

Data from the Longitudinal Aging Study Amsterdam (LASA) are not publicly available due to privacy and ethical restrictions, but are available upon reasonable request. Researchers can apply for access to LASA data through the LASA Steering Group (www.lasa-vu.nl). Requests for data access can be submitted via the LASA website and will be evaluated according to the LASA data access policy.
