# Supplementary material for: The association between total, animal-based, and plant-based protein intake and cognitive decline in older adults
Source: Eur J Nutr. 2025 Oct 6;64(7):288. doi: 10.1007/s00394-025-03810-x (PMC12500841; doi:10.1007/s00394-025-03810-x)
Supplement: Supplementary file 1 — Supplementary file1 [file 394_2025_3810_MOESM1_ESM.docx]

**Supplementary Table 1** Association between the source of protein (% energy) and level of cognitive function^a^ in Dutch older adults of the Longitudinal Aging Study Amsterdam (LASA).

|  |  |  | Model 1^b^  β (95%CI) | Model 2^c^  β (95%CI) | Model 3^d^  β (95%CI) |
| --- | --- | --- | --- | --- | --- |
| **Animal protein (% energy)** | Global cognition | Quartile 2  Quartile 3  Quartile 4 | -0.09 (-0.21,0.04)  -0.03 (-0.15,0.10)  -0.05 (-0.18,0.07) | -0.07 (-0.20,0.06)  -0.04 (-0.16,0.09)  -0.04 (-0.18,0.09) | -0.00 (-0.13,0.13)  0.00 (-0.13,0.13)  -0.01 (-0.12,0.14) |
|  | Information processing speed | Quartile 2  Quartile 3  Quartile 4 | -0.12 (-0.25,0.02)  -0.11 (-0.25,0.02)  0.01 (-0.12,0.15) | -0.09 (-0.22,0.05)  -0.08 (0.21,0.06)  0.06 (-0.08,0.20) | -0.09 (-0.22,0.05)  -0.07 (-0.21,0.06)  0.06 (-0.08,0.21) |
|  | Episodic memory | Quartile 2  Quartile 3  Quartile 4 | -0.07 (-0.20,0.05)  -0.11 (-0.24,0.02)  -0.11 (-0.23,0.02) | -0.07 (-0.19,0.05)  -0.11 (-0.23,0.01)  -0.10 (-0.22,0.03) | -0.08 (-0.20,0.04)  -0.12 (-0.24,0.00)  -0.11 (-0.23,0.02) |
|  | Executive function  ***Males*** | Quartile 2  Quartile 3  Quartile 4 | 0.07 (-0.08,0.23)  -0.05 (-0.20,0.11)  0.13 (-0.03,0.28) | 0.09 (-0.06,0.25)  -0.03 (-0.19,0.12)  0.14 (-0.03,0.32) | 0.08 (-0.07,0.24)  -0.05 (-0.20,0.11)  0.15 (-0.03,0.32) |
|  | ***Females*** | Quartile 2  Quartile 3  Quartile 4 | -0.13 (-0.27,0.02)  **-0.15 (-0.29,-0.00)***  **-0.14 (-0.28,-0.01)*** | -0.08 (-0.23,0.07)  -0.11 (-0.25,0.04)  -0.08 (-0.22,0.07) | -0.08 (-0.23,0.06)  -0.11 (-0.25,0.04)  -0.08 (-0.23,0.07) |
| **Plant protein (% Energy)** | Global cognition | Quartile 2  Quartile 3  Quartile 4 | -0.10 (-0.23,0.03)  0.06 (-0.07,0.18)  0.03 (-0.10,0.15) | -0.08 (-0.21,0.04)  0.05 (-0.08,0.18)  0.03 (-0.11,0.16) | -0.10 (-0.22,0.03)  0.04 (-0.10,0.17)  0.00 (-0.14,0.15) |
|  | Information processing speed  ***Males*** | Quartile 2  Quartile 3  Quartile 4 | -0.05 (-0.23,0.13)  0.01 (-0.18,0.19)  -0.09 (-0.27,0.10) | -0.10 (-0.27,0.08)  -0.04 (-0.23,0.14)  -0.16 (-0.35,0.04) | -0.10 (-0.28,0.09)  -0.05 (-0.24,0.15)  -0.16 (-0.37,0.06) |
|  | ***Females*** | Quartile 2  Quartile 3  Quartile 4 | 0.13 (-0.08,0.33)  0.08 (-0.11,0.28)  **0.24 (0.04,0.43)*** | 0.15 (-0.05,0.35)  0.11 (-0.08,0.31)  **0.30 (0.09,0.50)**** | 0.15 (-0.05,0.35)  0.12 (-0.08,0.32)  **0.31 (0.09,0.53)**** |
|  | Episodic memory | Quartile 2  Quartile 3  Quartile 4 | -0.07 (-0.19,0.06)  0.01 (-0.12,0.13)  -0.04 (-0.17,0.09) | -0.04 (-0.16,0.07)  -0.01 (-0.13,0.11)  -0.08 (-0.21,0.04) | -0.08 (-0.20,0.04)  -0.07 (-0.19,0.06)  **-0.16 (-0.30,-0.02)*** |
|  | Executive function | Quartile 2  Quartile 3  Quartile 4 | -0.06 (-0.17,0.05)  0.04 (-0.07,0.14)  0.01 (-0.10,0.12) | -0.04 (-0.14,0.07)  0.05 (-0.05,0.16)  0.02 (-0.10,0.13) | -0.06 (-0.17,0.05)  0.02 (-0.10,0.13)  -0.04 (-0.16,0.09) |

*^a^ Cognitive function level (z-score) differences are presented as unstandardized regression coefficients β (95% CI) for animal-based and plant-based protein intake quartiles Q2, Q3, and Q4, compared to the reference quartile, Q1. Animal-based protein intake quartiles (% energy) are defined as: Q1: <7.6, Q2: 7.6-9.2, Q3: 9.2-11.1, Q4: >11.1. Plant-based protein intake quartiles (% energy) are defined as: Q1: <5.2, Q2: 5.2-6.0, Q3: 6.0-7.2, Q4: >7.2. Negative β values indicate lower cognitive function levels relative to Q1. Results were stratified by sex in case there was significant interaction by sex (p ≤0.05). Sample sizes were 1339 for global cognition, 1300 for information processing speed, 1304 for episodic memory, and 1310 for executive function. Estimates in bold are statistically significant * p ≤0.05 ** p ≤0.01 *** p ≤0.001. ^b^ Model 1: adjusted for age, age2, educational level, and energy intake. ^c^ Model 2: additionally adjusted for sex (if no interaction), plant protein (% energy) (for animal protein model), animal protein (% energy) (for plant protein model), living status, number of chronic diseases, depressive symptoms, physical activity, smoking status, alcohol use, and Body Mass Index. ^d^ Model 3: additionally adjusted for diet quality (DHD-15,* range 0–130*).*

**Supplementary Table 2** The association between the source of protein (% energy) and cognitive decline^a^ with age in Dutch older adults of the Longitudinal Aging Study Amsterdam (LASA)

|  |  | **Animal protein**  **(% Energy)** |  |  | **Plant protein**  **(% Energy)** |  |  |
| --- | --- | --- | --- | --- | --- | --- | --- |
|  |  | Model 1^b^  β (95%CI) | Model 2^c^  β (95%CI) | Model 3^d^  β (95%CI) | Model 1^b^  β (95%CI) | Model 2^c^  β (95%CI) | Model 3^d^  β (95%CI) |
| Global cognition | Quartile 2 x age  Quartile 3 x age  Quartile 4 x age | 0.00 (-0.01,0.02)  -0.01 (-0.03,0.00)  -0.01 (-0.02,0.01) | 0.00 (-0.01,0.02)  -0.01 (-0.03,0.00)  -0.01 (-0.02,0.01) | 0.00 (-0.01,0.02)  -0.01 (-0.03,0.00)  -0.01 (-0.02,0.01) | -0.00 (-0.02,0.01)  -0.00 (-0.02,0.01)  0.00 (-0.01,0.02) | 0.00 (-0.01,0.02)  -0.00 (-0.21,0.01)  0.01 (-0.01,0.02) | 0.00 (-0.01,0.02)  -0.00 (-0.02,0.01)  0.01 (-0.01,0.01) |
| Information processing speed | Quartile 2 x age  Quartile 3 x age  Quartile 4 x age | -0.00 (-0.01,0.01)  **-0.02 (-0.03,-0.01)***  **-0.01 (-0.02,-0.01)*** | -0.00 (-0.01,0.01)  **-0.02 (-0.03,-0.01)***  **-0.01(-0.02,-0.00)*** | -0.00 (-0.01,0.01)  **-0.02 (-0.03,-0.01)***  **-0.01 (-0.02,-0.00)*** | 0.00 (-0.01,0.01)  0.01 (-0.00,0.02)  0.00 (-0.01,0.01) | 0.00 (-0.01,0.01)  0.01 (-0.00,0.02)  0.00 (-0.01,0.01) | 0.00 (-0.01,0.01)  0.01 (-0.00,0.02)  0.00 (-0.01,0.01) |
| Episodic memory | Quartile 2 x age  Quartile 3 x age  Quartile 4 x age | -0.00 (-0.01,0.01)  -0.00 (-0.01,0.01)  0.00 (-0.01,0.01) | -0.00 (-0.01,0.01)  -0.00 (-0.01,0.01)  0.00 (-0.01,0.01) | -0.00 (-0.01,0.01)  -0.00 (-0.01,0.01)  0.00 (-0.01,0.01) | -0.00 (-0.01,0.01)  -0.01 (-0.02,0.00)  -0.00 (-0.01,0.01) | -0.00 (-0.01,0.01)  -0.01 (-0.02,0.01)  -0.00 (-0.01,0.01) | -0.00 (-0.01,0.01)  -0.01 (-0.02,0.01)  -0.00 (-0.01,0.01) |
| Executive function | Quartile 2 x age  Quartile 3 x age  Quartile 4 x age | 0.00 (-0.01,0.01)  0.00 (-0.01,0.01)  -0.00 (-0.01,0.01) | 0.00 (-0.00,0.01)  0.00 (-0.01,0.01)  0.00 (-0.01,0.01) | 0.00 (-0.00,0.01)  0.00 (-0.01,0.01)  0.00 (-0.01,0.01) | 0.01 (-0.00,0.01)  0.01 (-0.00,0.02)  0.00 (-0.01,0.01) | 0.01 (-0.00,0.01)  0.01 (-0.00,0.02)  0.00 (-0.01,0.01) | 0.01 (-0.00,0.01)  0.01 (-0.00,0.02)  0.00 (-0.01,0.01) |

*^a^ Cognitive function decline (z-score) differences are presented as unstandardized regression coefficients β (95% CI) for animal-based and plant-based protein intake quartiles Q2, Q3, and Q4, compared to the reference quartile, Q1. Animal-based protein intake quartiles (% energy) are defined as: Q1: <7.6, Q2: 7.6-9.2, Q3: 9.2-11.1, Q4: >11.1. Plant-based protein intake quartiles (% energy) are defined as: Q1: <5.2, Q2: 5.2-6.0, Q3: 6.0-7.2, Q4: >7.2. Models test decline differences using interaction terms for protein x age. Positive β values for the protein x age interaction indicate slower decline with age relative to Q1. Sample sizes were 1339 for global cognition, 1300 for information processing speed, 1304 for episodic memory, and 1310 for executive function. Estimates in bold are statistically significant * p ≤0.05 ** p ≤0.01 *** p ≤0.001. ^b^ Model 1: adjusted for age, age^2^, educational level, and energy intake. ^c^ Model 2: additionally adjusted for sex, plant protein (% energy) (for animal protein model), animal protein (% energy) (for plant protein model), living status, number of chronic diseases, depressive symptoms, physical activity, smoking status, alcohol use, and Body Mass Index. ^d^ Model 3: additionally adjusted for diet quality.*

**Supplementary Table 3** Association between total protein intake (g/kg adjusted BW/d) and level of cognitive function^a^ in Dutch older adults of the Longitudinal Aging Study Amsterdam (LASA).

|  |  |  |  | Model 3^b^  β (95%CI) |
| --- | --- | --- | --- | --- |
| Global cognition | |  | Quartile 2  Quartile 3  Quartile 4 | -0.02 (-0.16,0.11)  0.02 (-0.13,0.16)  -0.03 (-0.20,0.15) |
| Information processing speed | |  | Quartile 2  Quartile 3  Quartile 4 | 0.01 (-0.13,0.16)  0.00 (-0.15,0.16)  -0.04 (-0.23,0.15) |
| Episodic memory | |  | Quartile 2  Quartile 3  Quartile 4 | 0.01 (-0.14,0.11)  -0.07 (-0.21,0.07)  **-0.21 (-0.38,-0.05)*** |
| Executive function | |  | Quartile 2  Quartile 3  Quartile 4 | -0.01 (-0.13,0.10)  -0.07 (-0.19,0.05)  0.02 (-0.13,0.17) |

*^a^ Cognitive function level differences (z-score) are presented as unstandardized regression coefficients β (95% CI) for protein intake quartiles Q2, Q3, and Q4, compared to the reference quartile, Q1. Protein intake quartiles (g/kg adjusted BW/d) are defined as: Q1: <0.9, Q2: 0.9-1.1, Q3: 1.1-1.3, Q4: >1.3. Negative β values indicate lower cognitive function levels relative to Q1. Sample sizes were 1339 for global cognition, 1300 for information processing speed, 1304 for episodic memory, and 1310 for executive function. Estimates in bold are statistically significant * p ≤0.05 ** p ≤0.01 *** p ≤0.001. ^b^ Model 3: adjusted for age, age^2^, educational level, energy intake, sex, living status, number of chronic diseases, depressive symptoms, physical activity, smoking status, alcohol use, Body Mass Index, and diet quality (DHD-15,* range 0–130*).*

**Supplementary Table 4** *Association between total protein intake (g/kg adjusted BW/d) and cognitive decline^a^ with age in Dutch older adults of the Longitudinal Aging Study Amsterdam (LASA)*

|  |  | Model 3^b^  β (95%CI) |
| --- | --- | --- |
| Global cognition | Quartile 2 x age  Quartile 3 x age  Quartile 4 x age | -0.00 (-0.02,0.01)  -0.01 (-0.02,0.01)  **-0.02 (-0.03,-0.00)*** |
| Information processing speed | Quartile 2 x age  Quartile 3 x age  Quartile 4 x age | -0.00 (-0.01,0.01)  -0.00 (-0.01,0.01)  -0.00 (-0.01,0.01) |
| Episodic memory | Quartile 2 x age  Quartile 3 x age  Quartile 4 x age | 0.01 (-0.01,0.02)  0.01 (-0.00,0.02)  **0.01 (0.00,0.02)*** |
| Executive function | Quartile 2 x age  Quartile 3 x age  Quartile 4 x age | 0.00 (-0.01,0.01)  0.00 (-0.01,0.01)  0.01 (-0.00,0.02) |

*^a^ Cognitive function decline differences (z-score) are presented as unstandardized regression coefficients β (95% CI) for protein intake quartiles Q2, Q3, and Q4, compared to the reference quartile, Q1. Protein intake quartiles (g/kg adjusted BW/d) are defined as: Q1: <0.9, Q2: 0.9-1.1, Q3: 1.1-1.3, Q4: >1.3. The model test decline differences using interaction terms for protein x age. Positive β values for the protein x age interaction indicate slower decline with age relative to Q1. Sample sizes were 1339 for global cognition, 1300 for information processing speed, 1304 for episodic memory, and 1310 for executive function. Estimates in bold are statistically significant * p ≤0.05 ** p ≤0.01 *** p ≤0.001. ^b^ Model 3: adjusted for age (centered at 55 years), age^2^, educational level, energy intake, sex, living status, number of chronic diseases, depressive symptoms, physical activity, smoking status, alcohol use, Body Mass Index, and diet quality.*
